# Predicting Thalassemia Using Feature Selection Techniques: A Comparative Analysis

**DOI:** 10.3390/diagnostics13223441

**Published:** 2023-11-14

**Authors:** Muniba Saleem, Waqar Aslam, Muhammad Ikram Ullah Lali, Hafiz Tayyab Rauf, Emad Abouel Nasr

**Affiliations:** 1 Department of Computer Science & Information Technology, The Government Sadiq College Women University Bahawalpur, Bahawalpur 63100, Pakistan; muniba@gscwu.edu.pk; 2 Department of Information Security, The Islamia University of Bahawalpur, Bahawalpur 63100, Pakistan; 3 Department of Information Sciences, University of Education Lahore, Lahore 54770, Pakistan; m.i.lali@ue.edu.pk; 4 Centre for Smart Systems, AI and Cybersecurity, Staffordshire University, Stoke-on-Trent ST4 2DE, UK; hafiztayyabrauf093@gmail.com; 5 Industrial Engineering Department, College of Engineering, King Saud University, Riyadh 11421, Saudi Arabia; eabdelghany@ksu.edu.sa

**Keywords:** thalassemia, classification, feature selection, filter-based, wrapper and embedded method

## Abstract

Thalassemia represents one of the most common genetic disorders worldwide, characterized by defects in hemoglobin synthesis. The affected individuals suffer from malfunctioning of one or more of the four globin genes, leading to chronic hemolytic anemia, an imbalance in the hemoglobin chain ratio, iron overload, and ineffective erythropoiesis. Despite the challenges posed by this condition, recent years have witnessed significant advancements in diagnosis, therapy, and transfusion support, significantly improving the prognosis for thalassemia patients. This research empirically evaluates the efficacy of models constructed using classification methods and explores the effectiveness of relevant features that are derived using various machine-learning techniques. Five feature selection approaches, namely Chi-Square (χ2), Exploratory Factor Score (EFS), tree-based Recursive Feature Elimination (RFE), gradient-based RFE, and Linear Regression Coefficient, were employed to determine the optimal feature set. Nine classifiers, namely K-Nearest Neighbors (KNN), Decision Trees (DT), Gradient Boosting Classifier (GBC), Linear Regression (LR), AdaBoost, Extreme Gradient Boosting (XGB), Random Forest (RF), Light Gradient Boosting Machine (LGBM), and Support Vector Machine (SVM), were utilized to evaluate the performance. The χ2 method achieved accuracy, registering 91.56% precision, 91.04% recall, and 92.65% f-score when aligned with the LR classifier. Moreover, the results underscore that amalgamating over-sampling with Synthetic Minority Over-sampling Technique (SMOTE), RFE, and 10-fold cross-validation markedly elevates the detection accuracy for αT patients. Notably, the Gradient Boosting Classifier (GBC) achieves 93.46% accuracy, 93.89% recall, and 92.72% F1 score.

## 1. Introduction

A series of hereditary blood diseases known as thalassemia are characterized by the abnormal or reduced production of one or more hemoglobin genes [1]. It ranks among the most common five birth complications [2]. There is a high prevalence of thalassemia worldwide, particularly in Southeast Asian nations. αT and βT are the two main classifications of defective globin [3]. Alpha-thalassemia may also result in hemoglobin H (HbH) disease, anemia, and hydrops fetalis syndrome. The amount of alpha-chain produced determines the disease’s severity. The major form of alpha-thalassemia has placed a heavy burden on society and harms the general population’s standard of living. Children with βT major experience impaired growth, hemolytic anemia [4], and aberrant development of the skeleton. For the remainder of their lives, the afflicted youngsters will require regular blood transfusions. Intermediary βT is less severe than βT major and may call for sporadic blood transfusions. Patients who depend on transfusions will experience an iron burden [5] and need chelation therapy to get rid of extra iron. Some young patients with βT major may benefit from bone marrow transplants [6]. Normal life expectancy is experienced by those who have the thalassemia trait. By the age of 30, βT major patients frequently pass away from cardiac problems brought on by iron overload.

The past couple of decades have witnessed a huge amount of study in machine learning. Novel ML algorithms can more effectively analyze medical data as a result of advances in the field and ongoing technological development. Researchers are creating stronger ML models to deal with progressively more complicated and substantial medical data thanks to this general rise. We are motivated to examine the development of ML as a thalassemia diagnostic tool in this paper. We can track the sort of change occurring in medical research by looking at the machine-learning methods applied to thalassemia prediction. We will scrutinize and estimate the empirical data obtained through ML techniques used to predict thalassemia trait (TT) in order to identify the most significant research trends in this field, highlight the challenges of using ML techniques for thalassemia prediction, point out the research gaps, lay the groundwork for future studies, and assess the impact of using machine learning on improving the outcomes of disease prediction. Additionally, this study might help researchers look into the unique ML approach applied to thalassemia applications throughout the last five years.

This paper is significant for ongoing research about the detection, diagnosis, and self-management of thalassemia. We review, analyze, and summarize approaches that pre-process and extract features from datasets related to thalassemia and use machine-learning algorithms to diagnose, classify, and detect it. These algorithms for thalassemia detection and diagnosis are evaluated using performance matrices. Future avenues for study will be determined by scientists working in the field. We aim to help academics, doctors, engineers, and administrators to create trustworthy and efficient data-driven interventions in the healthcare business. It is also aimed to signify the application of machine-learning research on real-world health applications. It is a comparative study that examines AI and ML methods for TT self-management, identification, and diagnosis. Initiation of this effort leverages investigation into the effectiveness of current ML approaches on publicly accessible thalassemia datasets.

The following research questions are most significant that our endeavor strives to answer:

R1 Which kind of datasets are utilized by ML-based prediction and management techniques for TT?
R2 Which ML methods are employed in the TT diagnostic?
R3 Which thalassemia variants can be detected using ML-based methods? Or what specific forms of thalassemia are being detected using ML-based methods?
R4 What standards are applied to evaluate ML classifiers for illness prediction?
R5 Which issues are addressed by ML-based applications in illness management and diagnosis?
R6 How effectively ML approaches will work with openly available datasets?

The most frequently used ML tasks and techniques, the effect of ML tasks and techniques on the performance of classification in thalassemia research, the overall performance of classifiers when using ML techniques, and comparisons of various classifier-preprocessing combinations in terms of accuracy rate are just a few of the issues we have addressed. Applications that patients can use to aid in diagnosis and management have been covered in detail. Additionally, we have looked at the main subtypes of thalassemia, diseases, and other detrimental health impacts associated with TDT. We also focused on ML approaches for assessing the health risks of thalassemia. This study is the first that we are aware of discussing TT diagnosis and management using ML and AI. It includes a systematic review of certain crucial aspects of the field, such as datasets (Table 1), ML applications for TT assistance, pre-processing, and feature extraction methods (Table 2, Table 3, Table 4 and Table 5), previously ignored by studies. As a result, efforts have been undertaken to investigate the body of research on ML approaches to TT diagnosis in the context of this study. The main contribution of the study is to provide new researchers with a baseline by evaluating the efficacy of models constructed using nine classification methods and exploring the effectiveness of relevant features (Table 6) that are derived using five feature selection approaches on two publicly available datasets. Previously, one study only used the iterative Chi-Square (Iχ2) [7] feature selection method, and another study used two techniques (i) Feature Reduction using Principal Component Analysis (PCA) and (ii) Singular Value Decomposition (SVD) [8]. Results of the experiments (Table 7, Table 8, Table 9, Table 10 and Table 11) show not only the comparison of selected feature sets with nine classifiers (Table 10) but also the effects of normalization and balancing using SMOTE (Table 11) presented for the evaluations. Lastly, the results of the experiments are also compared with previous approaches (Table 12).

## 2. Thalassemia

The generation of healthy alpha- or beta-globin chains, which make up hemoglobin, is impacted by a series of autosomal recessive hemoglobinopathies known as thalassemia. α- or β-globin chain [1,6] amalgamation problems may result in anemia, early oxidation of the blood, and inefficient erythropoiesis. Thalassemia patients may have extramedullary hematopoiesis and bone marrow enlargement as a result of chronic, severe anemia. Patients with microcytic anemia and normal or increased ferritin levels should be suspected of having thalassemia. Although genetic testing is necessary to confirm the diagnosis, hemoglobin electrophoresis can highlight shared traits across various thalassemia subtypes. Generally, thalassemia in carriers and trait states is asymptomatic.

Hydrops fetalis is a common birth defect brought on by alpha-thalassemia major. Beginning in early childhood (often before the age of two), βT major requires lifelong transfusions. Based on gene deletion or mutation, αT and βT intermedia present differently, and severe variants cause symptomatic anemia and need transfusions, whereas milder ones merely need monitoring. Transfusions, iron chelation therapy, hydroxyurea [9], hematopoietic stem cell transplantation [10], and Luspatercept [11] are all used in the treatment of thalassemia to reduce iron overload brought on by gastrointestinal absorption of iron, hemolytic anemia [12], and recurrent transfusions. Thalassemia consequences include perivascular iron deposition, bone marrow enlargement, and extramedullary hematopoiesis. A few of the morbidities that may arise from these issues include damage to the skeletal system, endocrine system [13], heart [14,15,16], and liver [5]. Life expectancy for people with thalassemia has greatly risen over the past 50 years thanks to better monitoring [5] of iron overload, increasing availability of transfusions of blood, and iron chelation treatment. Genetic counselling and screening in high-risk populations can reduce the prevalence of thalassemia [1]. Africa, India, the Mediterranean, Southeast Asia, and the Middle East [17,18,19] have the greatest rates of thalassemia prevalence. Preventative initiatives incorporating premarital and preconception counselling and testing may be contributing to a decline in incidence in these areas. Carriers of αT and βT make up around 5% and 1.5%, respectively, of the global population.

The globin chains in a physiological situation are a balanced mixture of α globin chains and non-α globin chains, primarily β-chains, which, when combined with α-chains, form adult hemoglobin (HbA), with δ-chains, form a minor portion of adult hemoglobin, called HbA2, or with γ-chains, form fetal hemoglobin (HbF). If one of the globin chains is not produced as much as it should while the other chains are still being produced normally, the developing red blood cell (RBC) will accumulate the other (unpaired) globin chains. In this manner, if α-gene is not produced in adequate quantities, an accumulation of β-gene will increase causing αT; likewise, if the production of β-gene chains declines, ultimately, accumulations in α-gene chains cause βT [20].

### 2.1. Alpha (α) Thalassemia

The term “alpha-thalassemia” (or “αT”) denotes a class of genetic blood illnesses categorized in a normal blend of β-globin chains [21] but diminished the creation of α-globin chains, which are both components of the hemoglobin molecule. Growing RBCs symbolize the buildup of unpaired globin chains. The formation of α-globin chains is regulated by four genes, two on each chromosome, implicating the possibility of several types of carriers.

#### 2.1.1. Silent Carrier

One (out of four) non-functional genes is present in a thalassemia alpha plus (α+) carrier [3], also referred to as αT minimal. Due to this, it may be very challenging to diagnose these carriers using a straightforward microscopic examination of their blood in a lab. These types of carriers can only be accurately identified through very specialized DNA analysis tests conducted in laboratories.

#### 2.1.2. Alpha Zero (α0) Thalassemia Carrier

Two (out of four) α-genes are either missing (deleted) or inactive. The two defective or deleted genes [22] might be situated either on the same chromosome (cis position) or on two distinct chromosomes (trans-position), depending on their specific location.

#### 2.1.3. Alpha (α) Intermedia Thalassemia

The condition identified as HbH ailment [23] is present when three α-globin genes are defective or absent, resulting in clinically significant anemia. This stops the additional α-chains from uniting with the α-globin chains to make common HbA, even if the α-globin genes are still completely functioning. Instead, a new hemoglobin (β4) called HbH is formed in the patient’s blood by joining the free-globin chains together. HbH can efficiently deliver oxygen to the tissues, just like common HbA, despite not being the hemoglobin typically found in human adult RBCs. Nevertheless, because of its relative instability, the molecule constantly breaks down, which results in premature red cell death or breakdown (hemolysis), which can cause mild to severe anemia in the affected person as well as other related health concerns such as splenic enlargement that ranges from mild to severe, tiredness, gallstone development, and deformed bones.

#### 2.1.4. Hb Constant Spring

Undetectable HbH, mutant allele causes a reduction in pf alpha globin activity Bart’s—Hydrops Fetalis [1]. This leads to no production of any α-chains, resulting in hemoglobin; a different type of hemoglobin termed Hb Barts (γ4) is created when free α-globin chains, which typically combine with α-globin chains to form the fetus’s hemoglobin (HbF), come together. Since this form of hemoglobin is unable to transport oxygen, life cannot be sustained by it [24]. Severe anemia brought on by this condition affects the unborn child and damages its heart.

### 2.2. Beta (β) Thalassemia

Minor, intermedia, and major are the three main types of βT [21,25].

#### 2.2.1. Beta (β) Thalassemia Minor

Caused by a mutation in one gene, they are formerly identified as “βT carrier” [26], or heterozygous βT”, and a majority of individuals have two different alleles.

#### 2.2.2. Beta (β) Thalassemia Intermedia

The mutation of two beta genes escalated thalassemia minor to thalassemia intermedia [27,28].

#### 2.2.3. Beta (β) Thalassemia Major

Two genes of the individuals defected with severe impairment in beta gene production are also known as “Cooley anemia” [29] and “Mediterranean anemia”. Like minor thalassemia, it has two different or multiple alleles of β0 or β+ genes. Balance in the globin chain is controlled by a specific form of beta gene modification. β0 means no generation of β-globin at all controlled by the defective allele. β++ denotes an allele with some residue beta globin generation (typically about 10%). The drop in the production of β-gene in β+ is minuscule. There are over 300 distinct βT alleles [30].

### 2.3. Other Variants of Thalassemia Carrier

One of the chromosomes that a person inherits from their mother or father is the only one that has a mutant gene [31]. They do not exhibit any clinical symptoms; thus, they do not need any kind of medical care or ongoing monitoring. They have some modifications in their RBCs, which are typically smaller and sometimes contain less hemoglobin, and are only detected by special blood tests but are not adequate to entail improvement.

Thalassemia can result in numerous types of disorders due to affected alleles, which might differ in their medical significance and requirement of blood transfusions. It comprises of two basic groups: one, TDTs that involve transfusion and two, NTDT [1,32] without the requirement of blood transfusion rendering to phenotyping. Without routine RBC transfusions, TDT patients would have numerous problems and have limited life expectancy. Patients with severe HbE/βT [33], βT major, HbH hydrops, or transfusion-dependent HbH illness, as well as those who have survived HbBart’s hydrops, fall into this group. For lifetime, the cornerstone of TDT care is transfusion therapy, while ineffective transfusion therapy might cause issues such as deprived development, deformities of face and bone or even making them fragile, spleen and liver enlargement, and everyday physical activity impairment.

Iron toxicity to vital organs is one of the foremost medical complications for thalassemia carriers. Higher intestinal absorption of nutritional iron and repetitive blood transfusions are the sources of iron accumulation. The iron content per unit of transfused blood is 200 mg, so patients who are regularly transfused develop iron overload [3,7]. Iron toxicity affects prime organs such as the liver and heart [8,9] and causes several endocrine disorders through the hypothalamus/pituitary axis, hypothyroidism, including growth obstruction, diabetes mellitus [34,35,36,37], and hypogonadism.

## 3. Systematic Literature Review

This portion of the article reviews five specific topics: databases, data preprocessing, the classification of thalassemia and health potential risks using ML, management applications based on ML, and performance metrics for assessing the success of the classification model.

### 3.1. Selection of Articles

Numerous attempts have been made to track down articles that use artificial intelligence and ML techniques for thalassemia research. The most prominent databases, including IEEE Xplore, ScienceDirect, and PubMed, were searched for a research paper on 8 May 2023. The fact that both databases contain a sizable collection of high-impact academic research publications in the fields of medicine and computer science serves as the main argument for their use. ML and AI are closely related to one another. Consequently, in scientific works, ML approaches are sometimes referred to as artificial intelligence approaches. Two searches using the terms “thalassemia” AND “Machine Learning” and “thalassemia” AND “Artificial Intelligence” are carried out to address this issue and to be more specific in discovering all pertinent papers. Our area of search is limited to publications published over the previous five years (2019–2023), which significantly dropped the collection to 113 (ML: 69, AI: 44) from the total 143 papers received from these searches (ML: 81 and AI: 162). A manual evaluation of each recovered document comes next. The main objective of this manual examination is to ascertain both the duplication of the article and its contribution to thalassemia research. Studies that do not use ML and AI methods are removed. The list is reduced by manual inspection to 39 papers.

### 3.2. Datasets Review

Researchers gathered most of the datasets used in the studies from their affiliated organizations or public health institutes, and a small portion utilized publicly available ones. The study analyzed CBC results that included age range, gender, and blood indicators such as RBC’s hemoglobin concentration, MCHC, MCV, and RDW. Considering the possibility of developing further diseases requires considering attributes such as family history, changes in urine color diabetes, spleen enlargement [21], and donors’ characteristics such as age and gender [22]. In addition, datasets are frequently divided as training and testing sets with ratios of 80:20 or possibly different ones such as a ratio of 70:30 or 50:50. Nevertheless, the work reported in [1] is deviated from this norm by employing two different datasets where one served as a test-bed for evaluating numerous classifiers, whereas another dataset served solely to evaluate the most successful classifier. Table 1 presents a complete breakdown of all essential data points and their respective features.

### 3.3. Preprocessing Techniques Review

The pre-processing of the dataset is carried out for a better representation to get distinct qualities. An overview of the preprocessing methods employed by researchers in a few specific academic papers is given in Table 2. Missing values and irrelevant characteristics are eliminated or managed in the data by using straightforward cleaning and normalization techniques [38,39]. SMOTE [39,40] is the sole method used for data balance, while Iχ2 [7] is a unique approach used for feature selection. Also, a combination of SVD [8] and PCA [41] as a feature reduction technique with data balancing technologies such as SMOTE and ADASYN is used. Filtering and thresholding, object detection, erosion and dilation, boundary detection, and lane extraction [42] are used for image datasets. DSIFT and DTL2 [43] are used for features of images.

### 3.4. Classifiers for Detection of Thalassemia

Thalassemia diagnostics might be more practical with the use of ML and quicker. The researchers employed a variety of ML algorithms to diagnose different variants of thalassemia or even discriminate alpha and beta variants from IDA. The goal of this part is to review the algorithms that are used in the key research mentioned. The next sections provide details of these algorithms. Table 3 provides an overview of the classifiers used by scholars in a few particular scholarly works.

**Table 1:** An overview of some key features used by thalassemia diagnosis (datasets and features).

Ref.	Description	Availability	Features
[7]	Two datasets used are homogenous with 159 records (87 females and 72 males) with ages over 18 years. The other dataset is heterogenous with a record of 1883 as 264 IDA, 27 βT & 1572 are normal.	Private	All CBC parameters, HbA, HbF, MCV, and MCH
[44]	Out of 3947 observations, 210 recordings of both classes (βT and non-βT) are taken in equal numbers to lessen bias. The mean age is around 25.	Private	Hb, MCV RBC count, PCV, MCH, RDW-CV, and MCHC
[8,38]	A total of 5066 individuals (53% males and 47% females) records are pulled from the PTPP database. Among them, 2015 are β-thalassemia carriers, while 3051 are β thalassemia non-carriers; 54% of the carriers are adults, and 46% are children.	Private	Age, Sex, Hb RBC, MVC, Hct, MCHC, MCH, PLT, RDW, and WBC
[45]	The dataset consisted of 594 cases (330 females and 264 males with average age of 29.7 years) with 229 healthy individuals, 160 patients with the α+-trail phenotype, and 205 individuals with a two-allele α-trail metamorphosis.	Private	Age, Gender, RBC count, Hct, Hb, MCH, MCV, RDW, and MCHC
[46,47,48,49,50]	The ten-variable dataset 150 has thalassemia, and 68 people are normal.	Private	Basophils, Monocytes, Eosinophils, Segment Neutrophils, Rod Neutrophils, Lymphocytes, PLT, Hb, HCT, and WBC
[40]	A total of 379 participants, including 79 positive cases of Thalassemia, made up the Kaggle dataset including a limited amount of data collected by Google form. https://www.kaggle.com/datasets/plenoi/thalassemia (accessed on 5 July 2023)	Public	Age, Gender, RBC, HGB, PCV, RDW, MCHC, PLT, TLC, MCV, and MCH
[51]	Out of the 350 patients at the Taipei Veterans General Hospital, 122 (34.8%) had no thalassemia variation, 179 (51.1%) had α-variant, and 49 (14%) had βT. Data are collected from January 2018 to January 2020.	Private	WBC, Hb, RBC, HCT, MCV, MCH, MCHC, RDW, PLT, RDW, RDWI, E&F, S&L, G&K, MDHL, MCHD, HH index, αT, and Βt
[52]	The hospital’s Laboratory Information System (LIS) is used to collect data on 1213 Chinese with low HbA2 levels from December 2018 to August 2020.	Private	RDW, Hct, MCV, RBC, Hbf, Hba, Hb, MCH, pregnancy, and age
[53,54]	Out of 342, only 152 individuals had thalassemia; data were collected from January 2016 to May 2019, in Elazig Public Health Laboratory. From January to July of 2018, 190 records (2 to 88 years) were declared as anemic.	Private	Hb, HCT, RBC, MCH, MCV, RDW, and MCHC
[55]	A total of 907 adults aged 18 and above were included in the study, and 59% had TT, while 41% had IDA.	Private	Hb, MCV, MCH, RDW, MCHC, and RBC
[39]	The study analyzed data on 45,498 individuals from 2012 to 2016 by the Palestine Avenir Foundation’s Thalassemia and Hemophilia Middle. 44,360 tests are normal, but 1138 individuals are confirmed to be carriers.	Private	CBC, RBC, Hct, Hb, MCV, MCHC, MCH, plt, RDW, and WBC
[56]	As per the opinion of doctors, tests are carried out on four types of data. To increase the precision level, it is vital to test the model with more genuine data.	Private	Hb, MCV, and MCH
[57]	CBC testing yielded results from a total of 750 tests conducted on individuals (males = 390 & females = 360) aged between 17 to 32 years old.	Private	RBC, Hb, HCT, and MCV
[58]	The dataset comprised a total of 8693 CBC test records along with 2918 genetic test data used for labeling type of thalassemia.	Private	HB, RBC, RDW, MCV, HCT, MCH, PLT, MCHC, WBC, patient’s sex and age
[59]	A dataset of 49 patients (31% high, 16% moderate, 10% low risk, and 43% zero signs) is collected from case files in the southwest section hospital of Nigeria.	Private	Age, gender, ethnicity, marital status, family history, social class, diabetes spleen enlargement, parent carriers, and urine color changes
[60]	Two datasets are involved as the first dataset had 6058 entities and the second, autonomous dataset had 2637 rows.	Private	Production machine, production date, Hb, Hct, extracted plasma, production site, donor sex, donor age, donor Hb, program and blood bag type
[61]	268 individuals	Private	RBC, HGB, MCV, and MCH
[42]	524 electrophoresis images	Private	Hb variants (HbA, HbA2)
[62]	From flow cytometry 302,652 cells and 3289 images	Private	Morphological
[63]	111 TDT patients in Iraq with an age range of 6 to 12 years	Private	Iron grade constraints (transferrin saturation percentage, iron, ferritin) and inflammatory (tumor necrosis factor-α and interleukin-1β)
[64]	Blood smears from 110 individual cases	Private	Morphological
[65]	1069 MRI images	Private	Morphological
[41]	Twenty blood smear images	Private	Morphological
[66]	7108 erythrocytes	Private	Shape, texture feature with gray level co-occurrence matrices (GLCM), color
[67]	Data collected from laboratories at Philippine General Hospital compares	Private	Perimeter (P) Area (A) Central Pallor (CP) Diameter (D) Deviation Value (DV) Target Flag (TF) Shape Geometric Factor (SGF)
[68]	Standing image datasets from Philippine General Hospital and Mendeley online.	Private	Codocytes and Elliptocytes
[43]	Disease-Specific Face (DSF) dataset totals 350 face images https://ieee-dataport.org/documents/disease-specific-faces (accessed on 5 July 2023)	Public	Morphological
[69]	1815 images of affected and normal blood cells https://www.kaggle.com/datasets/kmader/malaria-bounding-boxes (accessed on 5 July 2023)	Public	Morphological

**Table 2:** An overview of the existing preprocessing and feature selection techniques.

Ref.	Field of Use	Preprocessing
[38]	Classification of βT Carriers	Cleaning (missing value), data transformation (normalization), data reduction (attribute sub-selection)
[39]	Identifying βT carriers	Data cleaning (missing values) Normalization, balancing technique: SMOTE
[40]	αT Prediction	Data Balance: SMOTE
[7]	Differentiation of IDA and βT	Iχ2 feature selection
[8]	Prediction of βT	Data balance: SMOTE and ADASYN, Feature Reduction: PCA and SVD
[42]	Assessment of Thalassemia	Object detection, filtering and thresholding, erosion and dilation, boundary detection, and lane extraction
[41]	Classification of Thalassemia using fusion	PCA to eliminate feature redundancy
[43]	Facial diagnosis	DSIFT and DTL2

#### 3.4.1. Classifiers for Alpha thalassemia

To diagnose α+-thalassemia carriers, the DeepThal [45] framework uses 594 cases in total. The dataset consists of three classes: 205 individuals with a two-allele αT mutation, 160 individuals with α+-thalassemia, and 229 individuals who are healthy. As stipulated by CNN, accuracy is 80.77%, and sensitivity is 70.59%. The likelihood of thalassemia had been predicted using several well-known ML algorithms, [40] including LR, KNN, SVM, RF, Nave Bayes, Adaptive (ADA), Xgboost, DT, GBC, and MLP. SMOTE is used to balance the dataset. The ADA algorithm gave the greatest accuracy-related outcome, which is 100%, out of the ten algorithms. The SVM and Monte-Carlo cross-validation method [51] is used to distinguish between αT and βT. The dataset includes 350 registered patients from January 2018 to January 2020 at Taipei Veterans General Hospital, with 122 (34.8%) having non-thalassemia, 179 (51.1%) having αT, and 49 (14%) having βT. The SVM model outperformed all other indices with 0.76 AUC and 0.26 error rate on average. In terms of specificity (0.967), accuracy (0.915), PPV (0.942), AUC (0.948), and NPV (0.901), RF classifier [52] with the greatest overall performance outperformed seven equations in the independent test set. The technique is used to swiftly differentiate carriers of αT from those with low HbA2 levels. Thalassemia prediction is proposed by utilizing deep learning methods [58] utilizing genetic testing as the benchmark for performance. The thalassemia genetic test (2918) is the initial step. Identification of αT gene deletions serves as the inclusion criterion. The RBC indices from 8693 CBC tests, along with the patient’s age and sex, make up the other part. With 89.7% accuracy, the DNN model surpassed the statistical technique. All other characteristics—except RBC, HB, and MCV—are proven to be less significant than RDW and age.

#### 3.4.2. Classifiers for Beta Thalassemia

The Iχ2 feature selection method [7] is used for selecting 20 features out of 25 total given features of the dataset. On two datasets, 24 classifiers are applied, with the best accuracy of 97.48% obtained by Gaussian Support Vector Machine (MGSVM) on the first homogenous dataset of 159 and 99.73% with Coarse Tree (CT) on the second heterogeneous dataset of 1883. In India, βT diagnosis of expectant mothers is carried out utilizing three classifiers. To eliminate bias, use NB, C4.5 DT, and a back-propagation ANN [44] implementation in R Studio on a balanced number of selected βT and non-BTT individuals. C4.5 DT outperforms with an accuracy of 88.56% as opposed to ANN’s accuracy of 85.95% and NB’s accuracy of 82.49%. Rustam et al. [8] suggest a hybrid feature selection approach using SVD and PCA along with deep learning and supervised ML in several extensive trials. Data imbalance between carriers and non-carriers of βT is resolved by using ADASYN and SMOTE. The study employs multiple scenarios, such as the first one, which uses classifiers trained on the original dataset to discriminate βT carriers from non-carriers. The target variables in the second scenario are classified using ML models that have been trained on resample data. The third scenario combines SMOTE and ADASYN with two feature reduction strategies (PCA and SVA). The results of the experiments show that by combining SMOTE with the integrated framework of SVD and PCA, the proposed method beats the alternatives with a 0.96 accuracy score with RF. The three ML algorithms that make up the proposed SGR-VC [38] are SVM, GBM, and RF. Studies showed that the model, which utilized all RBC indices, has a 93% accuracy rate in identifying B-thalassemia carriers. A RF [46] method with 500 DT is recommended for precisely and thoroughly classifying thalassemia syndrome. As training data, the information from 150 thalassemia patients is separated into multiples of five ranging from 50% to 85%. With numerous ranges of training data, the algorithm has accuracy, recall, and precision are 98.99%, 100%, and 98.20%, respectively. Thalassemia diagnostic involves data from 150 individuals from Indonesia’s Hospital, with 10 attributes for SVM [47] base classification with a variety of kernel functions, i.e., polynomial, linear, and RBF. Gaussian RBF kernel gives 99.63% accuracy. By default, authors examine the normality of the case distribution concerning the characteristic using the Shapiro–Wilk method. Three situations are used to discriminate βT and IDA [53]. Both genders are tested individually in the other two situations, whereas they are evaluated jointly in the first scenario. SVM, ELM, KNN, LR, and RELM classification methods are used to classify each situation. Both the RELM and ELM algorithms produced an accuracy of 96.30% for female patients, 94.37% for male patients, and 95.59% when evaluating male and female patients simultaneously. TSVM [48] inspired by SVM is used to discover nonparallel hyperplanes to resolve a binary classification problem. Three commonly used kernels from earlier research are used to achieve this. RBF TSVM provided the most impressive results, as seen by its accuracy of 99.32%, precision of 99.75%, and f1 score of 99.24%. The least accurate TSVM, with an average recall of 99.79%, is polynomial. CART and BLTREED [55] are applied to the hematological parameters to separate βT and IDA individuals. The test dataset shows that for discriminating βT from IDA, CART outperforms BLTREED in terms of negative predictive value and sensitivity. Contrarily, CART has a high proportion of false positives. AUC results generally exhibited that the BLTREED model performed better. The density peaks (HCDP)-based hierarchical clustering [49] without and with kernel function is suggested for thalassemia identification. Some of these tasks include extracting the best clusters, calculating local density, and displaying a hierarchy. As a result, the polynomial kernel function is employed as the basis for the modification of this method. SVM [50] with grid search hyperparameter optimization is suggested to classify thalassemia data. RBF kernel-SVM gives more accuracy without optimizing hyperparameter. With holdout validation and 428.13 for C and 0.0000183 as gamma, the recommended approach produced 100% accuracy with 90% training data. Additionally, with C = 4832.93 and gamma = 0.0000183, it obtained 100% accuracy using 10-fold cross-validation. The results are noticeably superior to those obtained by applying the identical RBF kernel to an SVM with the default values give 73.33% accuracy, and with holdout plus 10-fold cross-validation, it goes to 57.14%. A hybrid data mining algorithm [39] is described for automatically detecting βT carriers using CBC test results of 45,498 patients. The put-forth identification paradigm involves two main steps. In the initial stage, the dataset’s significantly uneven class distribution is addressed using SMOTE oversampling. The next step is to train a collection of popular algorithms for classification, including DT, NB, MLP, and KNN. The NB classifier differentiates between carriers and non-carriers and βT the best at SMOTE 400% oversampling ratio. This blend has a 99.47% specificity and 98.81% sensitivity, respectively.

A fuzzy-based classification approach [56] is used to detect thalassemia using CBC data. This study discusses both model building and model software implementation. The findings of the CBC test, along with the hemoglobin levels, MCV, and MCH, are used to identify the type of thalassemia. Major, minor, intermedia, and normal are the four output models. The results are contrasted with opinions on thalassemia held by medical professionals to assess the model’s predictions against four data values. To verify that this model is accurate, further real-world data must be used. A novel technique [57] found on DHS is anticipated for the distinction of the βT and IDA. The method is successfully evaluated utilizing 132 CBC sample data that have been gathered. The most effective CBC indices are chosen to be used as the input of system using a PBIS approach. The results demonstrate that, with an accuracy of nearly 98%, the recommended strategy performs better than competing approaches in the literature. The current ANN, ANFIS, and MLP techniques, in that order, perform the best in terms of categorizing anemia. βT and IDA are distinguished from one another using ML techniques such as SVM and KNN on RBC indices [54]. The classifier’s input parameters are the RBC indices, and the performance of SVM and KNN is contrasted to determine which is more successful. Using ML algorithms with fewer input parameters results in higher performance. Two groups, one with 152 patients and the other with 190 patients that include both genders, make up the dataset. With the chosen settings, the accuracy rate in datasets of male and female rose from 95% to 95.3%. Alternatively, the NCA technique of component-based analysis feature selection is used to choose features from the datasets with an outstanding performance of 97% AUC. The distinction of IDA from βT is diagnosed by the ANN [61] technique. The dataset is obtained from 268 people’s CBC test parameters, where the diagnostic approach gives 92.5% accuracy, 92.33% specificity, and 93.13% sensitivity.

Automated evaluation of thalassemia has been studied using a unique deep-learning-based method [42] for thalassemia screening. The main goal of the project is to automatically obtain the tracks from electrophoresis envision strips and classify individuals as normal or abnormal with thalassemia. The suggested procedure involves database creation, lane extraction, object detection, and electrophoresis picture pre-processing. A thalassemia classification accuracy of 95.8% for the suggested technique is demonstrated using data from 524 cases. Score-CAM can be useful for understanding how the network decides as well as for boosting the end-user’s trust. Multilayer perceptron algorithms [62] potentially use cellular data from flow cytometry to predict specific cell genotypes. Particularly, the three potential MLP models perform well with 0.90 AUC in predicting FCD-HT cells. Meanwhile, the deep learning framework (T2D5) can also be suggestive of specific genotyping objectives when applied to DIC microscope pictures. Imagine that both tests can prove beneficial as additions to the genotyping techniques for modified cell lines that are already in use.

A typical screening approach for αT is the recognition of uncommon hemoglobin H (HbH) presence in RBCs. A convolutional neural network-based technique [64] is used to identify HbH. The method shows almost 91% sensitivity and 99% specificity for cells of HbH+ pictures taken at 40, 60, and 100 objectives. AI-based method with regard to a test set of 40 whole slide images (WSIs) demonstrated strong inter-rater reliability as well as increased specificity and sensitivity of slide-level categorization. Thalassemia is detected using both medical reports and blood smear images of patients. The blood analyzer extracts clinical data, while the CNN [41] extracts picture features from the blood smear image. Both landscapes are then integrated to create a meaningful feature set. Reduced computational complexity is achieved in this study by using PCA to eliminate feature redundancy. With the aid of integrated characteristics, thalassemic and normal patients are classified using classification methods including Naive Bayes, KNN, and RF, which achieved 99.1% accuracy and 100% specificity and sensitivity.

A novel AI-based system employs Deep Learning (DL) and an innovative combination of measures for diagnosing Thalassemia [70]. Several data engineering approaches, ranging from annotation of data to preparation, are utilized to create and evaluate a supervised semantic image segmentation model. To provide smoother and more precise predictions, transfer learning and Prediction Time Augmentation (PTA) are used. Quantitative findings revealed that 88% with PTA and 82% without PTA, respectively, represent the mean IoU score for predicting thalassemia. Results also indicated that the increases in thalassemia prediction when the total measure of loss scores falls.

Thalassemia peripheral blood smear images are segmented to create single erythrocyte sub-images. Morphological characteristics, such as distance angle signatures (DAS), moment invariants, cell, and central pallor geometry parameters, to improve the accuracy of erythrocyte categorization, morphological characteristics of the cell, including its core pallor, are paired with aspects of texture and color. Nine different erythrocyte morphologies that are found in thalassemia patients are classified using a multi-layer perceptron [66]. Based on the combination of attributes, the testing results using 7108 erythrocytes showed an accuracy of 98.11%.

Medical professionals such as technicians, hematologists, and pathologists identify RBC features including perimeter, area, shape geometric factor (SGF), target flag, diameter, and central pallor [67]. By identifying the edges and dividing overlapping cells, Sobel edge detection and watershed segmentation are effectively used to improve the picture for identifying RBCs. Inaccurate cell segmentation is still a problem with it. With the usage of a support vector machine, the result for categorizing RBCs nonetheless had a high accuracy of 93.33%. The physician in charge of the laboratories at Philippine General Hospital compares and assesses the data collected. Additionally, the method can link illnesses to detected aberrant RBCs. Codocyte and elliptocyte identification from blood smear images is automated using a Raspberry Pi [68]. The Elliptocytes and Codocytes in the PBS can be classified by the detection system using image with SVM. Codocytes and elliptocytes may be found in PBS pictures with an average classification accuracy of 94.31%. This will allow more investigations into the identification of aberrant RBCs and assist in locating early pathognomonic indicators of anemia and Thalassemia.

Deep transfer learning is used to distinctively recognize faces with thalassemia. Such a technique needs to be validated on single illnesses as well as on numerous diseases with healthy control. The two deep learning techniques of fine-tuning DTL1 and DTL2 [43] are employed for this purpose. DSIFT, a manually created feature, is used in comparison with using conventional ML techniques. The experimental findings of greater than 90% accuracy have demonstrated that CNN is the best appropriate transfer learning method for the brief dataset. Deep learning categories micrographs of malaria and anemia. Without using the conventional CBC test methodology, CNN [69] is used to process the images. Partially taken from the public domain and additionally gathered by the authors, data of 1815 images of effected and normal blood cells on a disc are preserved. The image pixels are multiplied by 255 to normalize the data, and the output is structured as a tensor (a vector). The developed model further tests on images to categorize them as normal blood cells, sickle cell anemia, thalassemia, malaria, and megaloblastic anemia, with a 93.4% accuracy.

#### 3.4.3. Classifiers for Risk Assessment of Thalassemia

Under the guidance of ML algorithms [59], a prediction model for the risk of thalassemia is developed with an accuracy of 94.12% using the factors and data that have been discovered and obtained. We run the thalassemia risk prediction model using the WEKA. Clinical criteria including household history, diabetes, enlarged spleen, color of urine, and parental carriers are also found using data of 51 people. Demographic parameters such as gender, age, marital status, ethnicity, and socioeconomic class are also detected. Risk is distributed as follows: 43% of instances are zero; 10% are low; 16% are moderate; and 31% are high. Eight [60] popular ML algorithms are tested against the first dataset to determine which one outperformed the others when repeated 50 times. These algorithms include MLR, NN, DT, SVM, RF, lgbmR, KNN, and RANSAC with a median MSE for Hb prediction of 3.89 and a 95% confidence interval of 3.3–4.5 (median R2 = 0.903, 95% confidence interval 0.885–0.921); MLR produced the best results. The two models (MLR with three and four features, respectively) with the optimal balance between complexity and performance are evaluated using the second 2637 dataset after retraining on the 6058 dataset.

Iron overload and immunological initiation should be treated to alleviate depression brought on by TDT, according to the nomological network incorporating experience, routes, and behavioral phenome manifestations [63]. This network also assesses overall cruelty and illness jeopardy and, as a result, forms a novel pharmacological target. Children with TDT (*n* = 111) and children in good health (*n* = 53) had iron status measures including iron, transferrin saturation percentage, ferritin, and inflammatory biomarkers like tumor necrosis factor measured and interleukin-1β, with the data analyzed using ML. TDT children with and without depression are differentiated using cluster analysis, which also identifies two subgroups of depressed children, one having a low sense of worth and the other who scored higher on social irritability. Four depressed crucial indications, key depressive, social irritability, physio somatic, and poor self-esteem, are confirmed as genuine constructs by exploratory. To accomplish unsupervised enactment of LIC (liver iron content) using five classes, four CNN models [65] 2D, 3D, LSTM of HippoNet-, and an ensemble HippoNet are employed. HippoNet-Ensemble outpaced the other networks in terms of accuracy and also outperformed HippoNet-LSTM in terms of sensitivity and specificity. Interobserver variability is 0.92 against 0.90 for multiclass accuracy. The summary of the thalassemia risk diagnostic used by researchers in a few articles is given in Table 3.

**Table 3:** An overview of Existing ML Classifiers used for Thalassemia Diagnosis (NA means information not available).

Ref.	Field of Use	Classifier	Compared with	Performance
[7]	Differentiation of IDA from βT	MATLAB (R2020a) classification learner toolbox	SVM, ANN, PCA and MLPs, ANFIS, MLPs, Math, Regular over-learning machine	SVM 97.48% accuracy with the 1st dataset & 99.73% accuracy with the 2nd dataset
[44]	Recognition of βT among antenatal women.	C4.5 DT, NB and ANN using R studio	Each selected classifier	Accuracy of 85.95% with ANN
[8]	Prediction of βT	DT, GBM, ADA, SVC, RF, ETC, LR LSTM, GRU, CNN, and CNN-LSTM.	Each selected classifier with different senarios	SMOTE with PCA and SVD had 96% accuracy
[45]	Prediction of the α+-T	CNN, SVM, MLP, RF, PLS, LR, ET, LGBM, XGB, DT, and KNNs implemented in Python	Each selected classifier	CNN accuracy of 80.77%
[38]	Classification of βT Carriers	SGR-VC ensemble (SVM, GBM, and RF)	Compared to each classifier in the model separately	93% accuracy
[46]	Classification of thalassemia	RF	RF with different percent of training data	98.99% accuracy
[40]	αT Prediction	KNN, NB, RF, LR, SVM, ADA Boosting, Xgboost, DT, MLP, and Gradient Boosting classifier using Google Colab	Each selected classifier	ADA accuracy 100%
[47]	Classifier for thalassemia	RBF, Polynomial, and linear kernel functions with SVM	SVM with different kernel and percent of training data	Gaussian RBF kernel with SVM accuracy 99.63%
[51]	Discriminating αT and βT	SVM with R.	SVM with 13 indices	NA
[52]	αT carrier discrimination	RF with R software version 3.6.2.	13 built ML models such as DT, KNN, SVM, ADA, LR, NB	Accuracy 91.5%
[53]	Discrimination of βT and IDA	LR, KNN, SVM, ELM, and RELM	DT, KNN, NB, DT, MLP, SVM, ANN, PCA, ANFIS, RBF, Math	96.30% accuracy for females, 94.37% for males, and 95.59% in co-evaluation of males and females with ELM and RELM
[48]	Thalassemia Classification	TSVM	TSVM with different kernel and percent of training data	99.32% accuracy
[55]	Finding of βT from IDA	BLTREED and CART	Each selected classifier	BLTREED model 96% accuracy
[49]	Thalassemia Classification	Based on density peaks, a hierarchical clustering algorithm with or without a kernel function	With different number of folds	NA
[50]	Thalassemia Classification	SVM with hyperparameter optimization using Grid Search	With different values of hyperparameter(C, gemma)	100% accuracy (90% training) gamma = 0.0000183 and C = 428.13
[39]	Identifying βT carriers	KNN, NB, DT and MLP in Weka (3.8.1)	Each selected classifier	99.71% Accuraccy
[56]	Prediction of thalassemia for children.	Fuzzy-based	NA	NA
[57]	Discrimination between IDA and βT	Dynamic Harmony Search (DHS).	ANN, ANFIS, SVM, KNN	Accuracy of approximately 98%
[58]	Predicting thalassemia	DNN model with 11 features then removing some of the 11 features	With different Combinations of featutes	89.7% accuracy
[54]	Discrimination of βT and IDA	KNN & SVM	ANNs, specialized ANNs Single Vector Analysis, MLP, LR, ANFIS, DT, NB, Neural Network, PBIS-ANN J48, mathematical method based on SVM and Heuristic algorithm	Accuracy 95.3%, Female, 94.5% Male
[59]	Thalassemia risk	NB and MLP in Weka	Each selected classifier	Accuracy 94.12% NB,100% MLP
[60]	Iron content and Hemoglobin Estimation	MLR, RF, KNN, DT, SVM, lgbmR, RANSAC and NN using Python	Each selected classifier with different features	NA
[61]	Discriminating between IDA and βT	ANN using MATLAB	MLP, SVM, KNN, RBF, PNN, ANFIS (radial basis function) Probabilistic neural network (*PNN*) adaptive network-based fuzzy inference system	92.5%, accuracy
[42]	Assessment of Thalassemia	MobileNetV2, InceptionV3, Densnet201, ResNet18, ResNet50, ResNet101, SqueezeNet	DT, ANN, KNN, SVM, Naïve Bayes, MLP, genetic programming	Accuracy 95.8% for InceptionV3
[62]	Predicting cell genotypes for βT	MLP, CRISPR genome editing technology.	NA	82% accuracy
[63]	Depression due to transfusion-dependent thalassemia	EFA and Cluster analysis,	NA	NA
[64]	Detecting approach for αT	Region-Based Convolutional Network (RCNN)	NA	Accuracy 97.6%
[65]	Liver iron content (LIC) evaluation	Deep-learning CNN—HippoNet-2D, 3D, LSTM, and HippoNet-Ensemble	NA	90% for multiclass accuracy
[41]	Classification of Thalassemia using fusion	RF, Naive Bayes, and KNN.	ANN, MLP, CNN, LR, PCA	Accuracy of 99.1%
[66]	Thalassemia diagnostic	MLP	With a combination of different features	Accuracy of 98.11%
[67]	Identification of Abnormal RBCs	SVM with Raspberry Pi	NA	Accuracy of 93.33%
[68]	Early indicators of Thalassemia.	SVM with Raspberry Pi	NA	Accuracy of 94.31
[43]	Facial diagnosis	CNN	CNN and SVM with different variation of features	90% accuracy
[69]	Thalassemia detection	CNN	NA	93.4% accuracy

### 3.5. Thalassemia Applications

The rule-based chatbot [71] for the management of βT endorses the outlook on health superiority that intends to improve patient confidentiality and timely care while addressing patient safety and efficacy. The chatbot offers accurate time for mandatory examinations and assessments, which can help to improve health outcomes and decrease the number of times patients need to see medical experts for checkups. Landbot is used to build the chatbot-based expert system. The chatbots were reviewed by 34 patients, the majority of whom (72%) found them simple to use, and more than 90% of them thought using them would be useful. To assist patients, doctors, and other healthcare professionals, an online specialist system [72] with a rapid response code is devised for βT administration. The overarching objectives are to promote patients’ lifetime healthcare and offer treatment suggestions.

Real-time patient information, including medical history, medication information, and appointment information, is provided via the system. Additionally, evaluated in real-world situations, it has been demonstrated to improve thalassemia management. For MHA (microcytic hypochromic anemia) patients, accurate classification between IDA and TT is critical. TT patients out of a total collection of 798 patients with MHA had a high number of TT (43.33%) and TT simultaneous with IDA (TT&IDA) patients (14.04%). To form a discriminant model, five ML algorithms are used: L-SVC, XGB, SVM, RF, and LR [73]. The information and links for the online thalassemia application are included in Table 4.

**Table 4:** An overview of existing Thalassemia management applications.

Ref.	Application	Purpose & Link
[71]	The rule-based chatbot	Thalassemia management support suggests exact scheduling for necessary tests and evaluations https://chats.landbot.io/v3/H-947072-772QZJR6XMJAGCJW/index.html (accessed on 5 July 2023)
[72]	Web-based expert system	Management of βT. Real-time patient information, including medical history, medication information, and appointment information. A QR code scanner or smartphone can decode the URL for each patient using a QR code.
[73]	Webpage tool of TT@MHA	Prediction based on patients’ provided parameters (RDW-SD, MCHCs, MCV, RBC, Hb, Age group, Sex, Pregnancy) https://dxonline.deepwise.com/prediction/index.html?baseUrl=%2Fapi%2F&id=26408&topicName=undefined&from=share&platformType=wisdom (accessed on 5 July 2023)
[74]	Web-based tool ThalPred	Prediction based on patients‘ provided parameters (Hb RBC, MCV Hct, RDW, MCHC, MCH) http://codes.bio/thalpred/ (accessed on 5 July 2023)

TT@MHA, with the RF model, gives better results, and the values for specificity, sensitivity, AUC, and accuracy are 91%, 91.91%, 94.2%, and 91.53%, respectively. The RBC indicators for differentiating TT from IDA are demonstrated using the interpretable rules developed from the RF model. Seven RBC parameters are used in an SVM model to construct a web-based utility called “ThalPred” [74]. AUC, MCC, and external accuracy of ThalPred’s predictions are 95.59%, 87%, and 98%, respectively. Without having to navigate the underlying mathematical and computational complexities, users may easily acquire the appropriate screening test result with ThalPred’s.

### 3.6. Performance Measures

Various performance indicators used in the selected research publications are shown in Table 5. Accuracy (Acc), specificity (Spec), precision, sensitivity (Sen), area under curve (AUC), F1-score, and positive predictive value are mostly used as performance measures.

**Table 5:** Performance analysis techniques used by researchers in the selected papers.

Acc	Recall	Precision/Positive Predictive Values	F1-Score	Sen	Spec	MCC	Negative Predictive Values	FPR	FNR	Youden’s Index	AUC	Ref.
✓	✓	✓	✓									[7,38]
✓				✓	✓						✓	[39]
✓	✓	✓	✓									[8]
✓		✓		✓	✓	✓	✓					[44]
✓				✓	✓	✓					✓	[45]
✓	✓	✓										[46]
✓	✓	✓	✓									[48]
			✓									[49]
✓												[50]
		✓		✓	✓		✓				✓	[51]
✓		✓			✓		✓				✓	[52]
✓		✓	✓	✓	✓							[53]
✓		✓	✓	✓	✓		✓	✓	✓			[54]
✓		✓		✓	✓		✓	✓	✓	✓	✓	[55]
✓		✓		✓	✓		✓					[57]
✓		✓	✓	✓	✓		✓			✓	✓	[58]
✓	✓	✓						✓				[59]
✓				✓	✓							[60,61,66]
✓	✓	✓									✓	[62]
				✓	✓							[63]
✓				✓	✓			✓	✓			[64]
✓						✓					✓	[74]
✓	✓		✓									Our method

In the majority of the papers, negative predictive values are also observed. Acc (22 times) and its combination with sensitivity and specificity are the performance metrics that are thought to be used by researchers most frequently. This combination is used 10 times. In six articles, the terms precision, accuracy, and recall are combined. The Matthews correlation coefficient (MCC) (two times), FPR (four times), FNR (three times), Youden’s index (two times), and positive predictive value (in three articles) are other performance matrices that are not frequently used by scientists.

The first section of this paper includes a systematic review of AI-based and ML-based thalassemia diagnostic methods. The IEEE Xplore, ScienceDirect, and PubMed databases are used to choose the primary literature. Additionally, two search phrases are employed to narrow down the pool of quality primary research papers for this analysis and fewer skewed selection studies. A rigorous screening resulted in the selection of 39 research papers for this study. This analysis focuses on five particular topics: databases, data preparation, the classification of thalassemia and health threats using machine learning, management applications based on ML, and measures of performance for evaluating the effectiveness of the classification model. In the chosen studies (Table 1), researchers employed either privately developed, exclusive datasets, or open-access datasets. Numerous scholars have developed their individual distinct (Self-compile) datasets in several studies using data that they received from a specific system or hospital.

According to our study, several experiments are carried out, each using a distinctive dataset. These trials do, however, endure two critical shortcomings. Initially, the established models of classification concentrate on a certain modality, using data that are taken from a single hospital and processed using a single instrument. Consequently, the categorization model developed from the data gathered could not be applied on a bigger scale. There is a lot of diagnostic equipment available nowadays that gathers TT data, which is the cause of this. Each system may have a standard that includes a range of characteristics and conditions. It is suggested that data should be acquired from a variety of clinics and a variety of diagnostic tools. The classification model produced by such a multimodal dataset may be used on a larger scale and is more trustworthy. Second, just a few features are available in special databases. Due to over- or under-fitting, the described classification model suffers. Therefore, using a structured, easily available TT dataset is a smart move to treat TT conditions in its early stages. The majority of researchers evaluated the effectiveness of their classifiers using accuracy, specificity, and sensitivity. This blend is frequently used for TT prediction using ML approaches. Accuracy, specificity, sensitivity, and precision are other fusions that are frequently used by the research community. The analysis of data preparation, normalization, feature selection, and ML classification approaches is covered in the next section of the study with a working example. Nine ML basic classifiers are used with two public datasets from Kaggle [75] in the experiment.

## 4. Material and Methods

**Figure 1:**
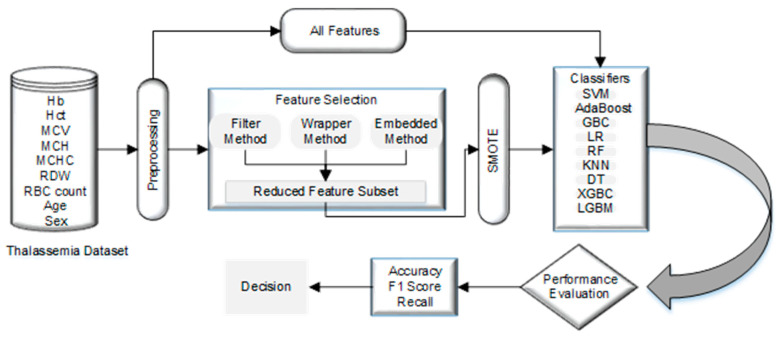
Our proposed Methodology.

This section examines normalization, resampling, nine classification and five feature selection techniques on two public datasets. Figure 1 illustrates the steps of the used methodology in detail.

### 4.1. Dataset

We have used two datasets. The first dataset is taken from Kaggle [75], which contains records of 616 thalassemia patients. It has 13 features, 387 of which are classified as thalassemia and 229 as normal. The features of the dataset include CBC parameters and indices, including Hb concentration, MCV, Hct, MCHC MCV, MCH, RBC count, RDW, and more. One of the reasons for selecting this public dataset is that mostly reported works use these features and the total number of features are close to the dataset with highest number of features [51]. Diagnostic attributes for the desired variable are found in the dataset and include both normal and αT carriers. The dataset contains 56% females and 44% meals with two main classes as one normal and other αT trail (Alpha-thal-1, Alpha-thal-2, and HbH disease). The second dataset is also taken from Kaggle [76], which contain records of 203 individuals of both genders. It has 15 features (Hb, PCV, RCB, MCV, MCH, MCHC, RDW, WBC, Neut, Lymph, PLT, HBA, HBA2, HBF, and Sex), 55 of which are normal and 148 alpha carrier.

### 4.2. Data Preparation

Data might include noise, consistency issues, and incompleteness since information is typically gathered from several sources. These traits may produce incorrect results. This issue may be resolved by preprocessing the dataset before applying classification models to enhance the accuracy of the classification process.

#### Data Cleaning

For datasets to manage missing values and incorrect inputs, data cleaning is a crucial step that must be undertaken. Thus, addressing missing numbers and eliminating discrepancies may aid in enhancing the quality of data for subsequent use. The process of cleaning the data is initiated first. A duplicate value along with null value checks performed on the dataset. The dataset is then checked to determine whether there are any noisy values. The dataset has also had inadequate features eliminated since they had no bearing on the classification outcome, such as SEA-THAI, which is only used to identify deletions of the Southeast Asian and Thai patients. 3.7/4.2, ETC, and CS/PS are additional qualities that are eliminated.

### 4.3. Normalization

Before training any classifier, normalization is a crucial data mining procedure that should be used. The goal is to ensure that all characteristics have a similar variety of values and to prevent the training process from being impacted by attributes with a broader range of values. In this study, the normalization method described in Equation (1) is used to normalize all numerical characteristics to the range [0, 1].

(1) $$Normalization(e_{i})=\frac{e_{i}-E_{min}}{E_{max}-E_{min}},$$

where $E_{max}$ and $E_{min}$ stand for the feature’s maximum and minimum values, respectively.

### 4.4. Using SMOTE to Address the Unbalanced Data Issue by Data Resampling

When one class of instances dominates the dataset by a large margin over the other classes, the dataset is said to be unbalanced [40]. The majority class in an unbalanced dataset is distinguished from the minority class by the number of occurrences; in an unbalanced dataset, the majority class has a greater number of instances [77]. Unbalanced datasets present a significant problem when training classification models [78]. This is because the most common classification algorithms prioritize accurately classifying the main class to make the most of inclusive classification accuracy while neglecting occurrences of the minor class, which are frequently more significant. When compared to random oversampling, SMOTE does not duplicate existing data entries but instead creates new synthetic data for the minority sample [79]. SMOTE is a potent and popular oversampling technique that is frequently used in literature to address the problem of unbalanced data. Various medical research projects have recently utilized SMOTE [39,40,80]. More specifically, SMOTE determines the k examples that are physically nearby to the minority example for each occurrence in the minority class. The usual Euclidean distance is used to determine this distance. The next phase is the generation of fresh synthetic samples.

### 4.5. Feature Selection

Increasing prediction accuracy while maintaining the diversity of features is difficult. Therefore, before using an ML model to predict outcomes, a feature selection procedure should be carried out to choose important features from the original feature set. The selection procedure used for features also enhances the performance of ML models by lowering the amount of time required to compute and the issue of over fitting. The information might not be sufficient to create predictions if we simply choose a few attributes to provide as input for an ML model. The dimensionality curse causes the generalization performance to suffer when there are a lot of features since it prolongs execution time. To make accurate forecasts, only the factors that have the most effects on the outcomes should be chosen. The current survey article covers numerous kinds of feature selection [81,82] methodologies together with their distinct selection criteria for the pertinent aspects of standard data. In this work, we have used five variants of three well-known approaches [83,84] that are one, Linear Regression Coefficient, then RFE using Tree [85] and Gradient-Based Estimators from embedded features; χ2 [86] from the filtering approach; EFS from the Wrapper approach [87]. Details of the features with selected features are shown in Table 6 for both datasets.

**Table 6:** Feature selection methods and feature sets.

Feature Selection	Feature Set (First Dataset)	F#	Feature Set (Second Dataset)	F#
Feature importance using RF	MCV, MCH, RDW	F11	Hb, PCV, RBC, MCV, MCH, WBC, Lymph, HBA2	F21
Feature importance using GBDT	Age, Hb, MCV, MCH, RDW, RBC count	F12	Hb, PCV, MCV, MCH, MCHC, RDW, WBC, Neut, Lymph, PLT, HBA2, HBF	F22
Estimation of coefficients using linear regression	Sex, Hb, MCH, RDW	F13	Hb, RBC, HBA2, HBF, Sex	F23
χ2	MCV, MCH, RDW, Hb, RBC count, Hct	F14	PLT, lymph, MCV, MCH, Neut	F24
	MCV, MCH, RDW, ‘Hb	F15	PLT, lymph, MCV, MCH	F25
	MCV, MCH	F16	PLT, lymph, MCV	F26
Exhaustive Feature Selection (EFS)	Age, Sex, Hb, MCV	F17	Hb, PCV, RBC, MCV	F27

#### 4.5.1. Filter-Based Feature

One filter-based feature selection approach, namely Chi-square, is primarily used in the proposed study. Using this function “weeds out”, the characteristics are most likely to be class autonomous and so insignificant for sorting since the χ2 test detects dependency between stochastic variables. Following is a list of the steps that make up this procedure. All of the characteristics from the original dataset should first be selected. Then, employing the χ2 function from the scikit-learn, its score for each characteristic is calculated using Equation (2).

(2) $$\chi 2=\sum \frac{{\left(f_{o}-f_{e}\right)}^{2}}{f_{e}},$$

where $f_{e}$ denotes the anticipated frequency and $f_{o}$ denotes the observer. To build a model, due to its greater dependence on the target feature, the feature with the greatest χ2 value is picked. For experiment purposes, three set of highest value features are selected.

#### 4.5.2. Wrapper Methods

This approach primarily employs a searching approach to estimate the variable subsets of autonomous attributes $S^{\prime }\subseteq S$ by giving $S^{\prime }$ as input to the selected algorithm and then measuring the efficiency. The techniques are continued until the required suboptimal subsets are identified when the cardinality of features in a dataset is N, in which case $2^{N}$ subsets are viable.

The process to choose the finest feature subgroup uses EFS with random forest. The first step is the selection of all the characteristics from the original dataset. Secondly, initialize the four minimum and five maximum features variables to begin the feature selection process. Repeat the process with different values.

#### 4.5.3. Embedded Methods

The filter and wrapper techniques are combined in this hybrid approach. The algorithms also include their method for choosing features in this section. These assist in creating the ideal subset and providing it to the training model. Algorithms play a role in the development of embedded feature selection techniques. Linear Regression Coefficient, RFE with Tree and Gradient-Based Estimators, are used for feature selection.

### 4.6. Classification Model

Nine well-known classification methods are used in the classification process to predict thalassemia. The chosen algorithms include KNN (K-Nearest Neighbors), DT (Decision Tree), GBC (Gradient Boosting Classifier), LR (Logistic Regression), ADA (AdaBoost), XGB (Extreme Gradient Boosting), RF (Random Forest), LGBM (Light Gradient Boosting Machine), and SVM (Support Vector Machine). The majority of the algorithms are fairly simple to use and use and are often utilized in earlier studies in the same field. In our study, we picked a standard application for these methods, which might be highly useful for academics and experts to replicate our results and compare them. Finding the prediction technique with the maximum generalization performance is the goal of this step.

### 4.7. 10-Fold Cross-Validation

One of the approaches for classification validation is K-fold cross-validation. By randomly dividing our dataset into other groups, we can validate our findings. In this, one set is utilized for training and the other, K-1 set, for validation. With 10-fold cross-validation, we will now verify our result. The dataset is mixed up and divided into 10 sets, and one set is chosen for validation, while the other four are used for training.

## 5. Result

The tests conducted per technique are shown and discussed in this section. We consider four experimental circumstances; the details and results from the first dataset are shown in Table 7 and Table 9. A comparison of both datasets with feature selection and preprocessing is presented in Table 10 and Table 11. In the first two experiments, the conventional classifiers KNN, DT, GBC, LR, ADA, XGB, RF, LGBM, and SVM are used with and without feature selection. In the next two experiments, oversampling, normalization, and 10-fold are involved with the identical classifiers discussed in the previous scenario on the newly resampled data.

We employ the most popular assessment measure used in the literature for medical applications to assess the effectiveness of classification models. These measures include classification accuracy, F1 score, and recall. Each research investigation divides the dataset as 80:20 for the classification algorithms. The testing set is used to evaluate the models after they have been developed using the training set.

**Table 7:** A performance comparison of the classifiers and the feature selection methods (first dataset).

Features	Measures (%)	SVM	SVM Hyperparameter (C, gamma, kernel = ‘rbf’)	GBC	ADA	LR	DT	XGBC	RF	LGBM	KNN *n* = 3	KNN *n* = 6
* C = 100, gamma = 0.0001												
All features (First dataset)	Accuracy	83.87	86.29	85.48	83.87	88.31	83.12	87.01	88.31	85.71	85.71	87.66
	F1 Score	82.55	84.607	83.6	82.19	88.02	82.88	86.74	88.02	85.3	85.42	87.33
	Recall	84.82	85.3	84.05	83.51	88.19	83.52	87.08	88.19	85.3	85.74	87.41
* C = 10, gamma = 0.01												
F11	Accuracy	83.87	87.1	89.52	86.29	84.42	82.47	86.36	82.47	85.71	85.06	85.71
	F1 Score	82.56	85.6	88.23	84.95	83.51	80.99	85.32	81.39	84.79	84.35	84.68
	Recall	84.82	86.55	88.99	86.61	84.65	81.11	85.75	82.32	85.66	83.84	83.72
* C = 100, gamma = 0.0001												
F12	Accuracy	83.06	87.1	85.48	80.64	90.91	90.9	88.96	88.31	86.36	85.71	83.76
	F1 Score	81.77	85.6	83.97	78.64	89.63	89.52	86.74	86.22	83.62	82.95	80.25
	Recall	84.23	86.55	85.36	79.82	90.07	89.52	85.37	85.44	82.38	81.9	78.84
* C = 1000, gamma = 0.0001												
F13	Accuracy	84.68	86.29	86.29	84.68	86.36	79.22	79.22	84.42	78.57	81.17	84.42
	F1 Score	82.1	84.61	84.61	82.1	85.93	78.51	78.4	83.62	77.67	80.26	83.62
	Recall	84.11	85.3	85.3	84.11	87.36	79.58	79.24	84.09	78.38	80.8	84.09
* C = 10, gamma = 0.001												
F14	Accuracy	83.06	86.29	87.01	84.68	91.56	82.47	84.42	87.01	83.77	84.42	85.06
	F1 Score	81.77	84.78	85.6	83.18	91.04	80.31	83.03	85.86	82.01	82.6	83.38
	Recall	84.23	85.95	86.55	84.76	92.65	79.69	83.31	86.17	81.96	82.04	82.96
* C = 10, gamma = 0.001												
F15	Accuracy	83.87	86.29	88.71	86.29	85.71	83.12	88.31	87.01	85.06	83.12	83.76
	F1 Score	82.56	84.78	87.4	84.95	84.89	82.45	87.55	86.35	84.25	82.14	82.65
	Recall	84.82	85.95	88.39	86.61	84.51	82.64	86.93	86.14	83.97	81.79	82.05
* C = 1000, gamma = 0.01												
F16	Accuracy	83.87	84.68	83.87	88.71	87.66	77.92	80.52	81.89	82.47	84.42	85.06
	F1 Score	82.56	82.8	81.55	87.4	86.61	76.15	78.95	80.5	81.13	82.88	83.06
	Recall	84.82	83.45	81.55	88.39	86.76	76.36	79.19	81.01	81.51	82.63	81.92
* C = 1000, gamma = 0.0001												
F17	Accuracy	82.26	86.29	84.68	79.84	88.31	83.12	83.12	87.01	84.42	83.12	87.01
	F1 Score	80.99	84.95	82.99	78.84	87.71	82.02	82.35	86.5	83.8	82.25	85.85
	Recall	83.63	86.61	84.18	82.5	88.24	82.02	83.05	87.54	84.77	82.7	85.15

* Value of SVM Hyperparameter (C, gamma).

**Table 8:** A performance comparison of the classifiers and the feature selection methods (second dataset).

Feature	Measures	SVM	SVM Hyperparameter (C, Gamma, Kernel = ‘rbf’)	GBC	ADA	LR	DT	XGBC	RF	LGBM	KNN *n* = 3	KNN *n* = 6
* C = 0.1, gamma = 1												
All features (second dataset)	Accuracy	73.17	73.17	70.73	63.41	66.67	64.71	70.59	64.71	70.59	82.35	80.39
	F1 Score	42.25	42.25	57.29	38.81	45.02	61.36	62.22	47.84	62.22	62.32	52.78
	Recall	50.0	50.0	56.96	43.33	52.78	61.36	62.12	52.52	62.12	61.11	52.78
* C = 10, gamma = 0.01												
F21	Accuracy	73.17	73.17	63.41	58.54	54.90	52.94	54.90	50.47	58.82	49.02	52.94
	F1 Score	42.25	65.85	44.34	45.59	35.44	49.67	46.71	44.36	51.34	36.00	34.62
	Recall	50.0	50.17	46.21	45.76	48.28	50.39	50.47	52.82	54.46	43.65	46.55
* C = 10, gamma = 0.1												
F22	Accuracy	73.17	73.17	75.61	70.73	66.67	58.82	64.71	72.55	66.67	68.73	70.59
	F1 Score	42.25	42.25	66.94	60.32	40.00	47.11	44.02	48.11	49.03	53.64	41.38
	Recall	50.0	50.0	66.06	59.85	45.95	47.20	46.81	52.22	50.39	53.96	48.65
* C = 0.1, gamma = 1												
F23	Accuracy	73.17	73.17	60.98	70.73	72.55	68.62	64.71	72.52	74.51	72.54	74.50
	F1 Score	42.25	42.25	52.87	64.66	48.11	53.62	50.96	56.46	63.51	59.43	49.19
	Recall	50.0	50.0	53.18	65.61	52.22	53.95	51.25	56.66	62.45	58.88	53.57
* C = 0.1, gamma = 1												
F24	Accuracy	73.17	73.17	75.61	68.29	72.55	62.75	62.75	64.71	70.59	68.63	72.55
	F1 Score	42.25	42.25	61.17	51.76	42.05	43.03	38.55	39.29	47.06	46.03	42.05
	Recall	50.0	50.0	60.30	52.42	50.0	45.46	43.24	44.59	50.87	49.52	50.0
* C = 0.1, gamma = 1												
F17	Accuracy	73.17	73.17	70.71	65.85	88.23	64.70	70.59	76.47	74.51	82.35	80.39
	F1 Score	42.25	42.25	57.29	45.64	46.88	44.02	55.03	50.32	54.12	60.33	52.78
	Recall	50.0	50.0	56.97	47.88	50.0	43.88	61.67	50.56	56.67	61.11	52.78

* Value of SVM Hyperparameter (C, gamma).

**Table 9:** Performance of Classifier with (**a**) SMOTE and 10-fold (**b**) with Normalization, SMOTE, and 10-fold (first dataset).

Feature	Parameter and Value (%)		Classifier	Parameter and Value (%)		Classifier
	(a) SMOTE and 10-fold			(b) Normalization, SMOTE and 10-fold		
F11	Accuracy	91.03	ADA	Accuracy	91.02	GBC
	F1 Score	91		F1 Score	91.31	
	Recall	91.67		Recall	93.89	
F12	Accuracy	93.46	GBC	Accuracy	91.03	GBC
	F1 Score	93.65		F1 Score	91.21	
	Recall	95.46		Recall	94.03	
F13	Accuracy	89.82	GBC	Accuracy	91.65	GBC
	F1 Score	89.53		F1 Score	91.73	
	Recall	87.92		Recall	94.03	
F14	Accuracy	86.25	KNN (*n* = 3)	Accuracy	89.23	LGBM
	F1 Score	87.63		F1 Score	89.47	
	Recall	95.14		Recall	91.53	
F15	Accuracy	89.23	XGBC	Accuracy	86.84	XGBC
	F1 Score	89.71		F1 Score	86.73	
	Recall	93.06		Recall	86.67	
F16	Accuracy	90.48	DT	Accuracy	88.64	DT
	F1 Score	90.73		F1 Score	88.6	
	Recall	91.81		Recall	88.19	
F17	Accuracy	90.51	GBC	Accuracy	92.79	RF
	F1 Score	90.04		F1 Score	92.68	
	Recall	88.19		Recall	93.89	

**Table 10:** A performance comparison of classifiers on first and second datasets with various features.

Features	Accuracy (%)	Classifier	Feature	Accuracy (%)	Classifier
First Dataset			Second Dataset		
All Feature	88.31	LR	All Feature	82.35	KNN *n* = 3
F11	89.52	GBC	F21	73.17	SVM
F12	90.91	LR	F22	75.61	GBC
F13	86.36	LR	F23	74.50	KNN (*n* = 6)
F14	91.56	LR	F24	75.61	GBC
F17	88.31	LR	F25	88.23	LR

**Table 11:** A performance comparison of classifiers on first and second datasets with Normalization, SMOTE, and 10-fold.

Features	Accuracy (%)	Classifier	Features	Accuracy (%)	Classifier
First Dataset with Normalization, SMOTE and 10-Fold			Second Dataset with Normalization, SMOTE and 10-Fold		
F11	91.02	GBC	F21	85.0	RF
F12	91.03	GBC	F22	90.0	ADA
F13	91.65	GBC	F23	83.33	SVM
F14	89.23	LGBM	F24	86.67	GBC
F17	92.79	RF	F25	86.67	RF

In this procedure of comparison analysis, Python is the programming language utilized to create the analytical model on Google Code lab. There are 616 samples in the repository, 229 of which are normal, and 387 of which are positive for thalassemia. Five feature selection algorithms, including χ2, EFS, RFE by using tree-based and gradient-based, and Linear Regression Coefficient are used to choose the best feature subsets for classification. Nine models as KNN with *n* = 3 and *n* = 6, DT, GBC, LR, ADA, XGB, RF, LGBM, and SVM simple also with hyperparameter tuned with different generated values of C, gamma, and kernel = ‘rbf’ are used to evaluate the performance.

### 5.1. Experiment I: Classification without Feature Selection

This experiment evaluates each of the aforementioned techniques for classification without using the feature selection approach on the dataset previously assembled. Results of all the classifiers are available in Table 7. The original feature set used the LR classifier to obtain maximum accuracy of 88.31%, recall of 88.19%, and f-score of 88.02% with first dataset. KNN shows the highest accuracy of 82.35%, recall of 61.11%, and f-score of 62.32% with second dataset (Table 8).

### 5.2. Experiment II: Classification with Feature Selection

We initially use the feature selection strategy on the first dataset, followed by classifiers, to elevate the classification models generalization potential for identifying αT patients (Table 7). Six characteristics, including MCV, MCH, RDW, Hb, RBC count, and Hct, are chosen by χ2 to provide the greatest accuracy of 91.56%, recall of 91.04%, and 92.65% f-score with LR. With Age, Sex, Hb, and MCV as inputs, EFS and LR yield a maximum of 88.31% accuracy, 87.71% F1-score, and 88.24% recall. GBC and tree-based RFE (Age, Hb, MCV, MCH, RDW, RBC count) achieved accuracy, F1-score, and recall of 89.52%, 88.23%, and 88.99%, respectively. Age, Hb, MCV, MCH, RDW, and RBC count are used as features to obtain maximum accuracy when gradient-based feature selection and LR. Finally, the Linear Regression Coefficient with four features (Sex, Hb, MCH, and RDW) obtained by LR estimation of coefficients offers 86.36% accuracy, 85.93% f-score, and 87.36% recall. LR shows accuracy of 88.23% (Table 8) with second dataset with four attributes (Hb, PCV, RBC, and MCV) given by EFS. All models built from the chosen subsets of features using various feature techniques perform better than the feature subsets from the original first dataset, according to the analysis of findings (Table 7). However, for the second dataset, only EFS as result is presented in Table 8.

### 5.3. Experiment III: Classification with SMOTE, Feature Selection, and 10-Fold Cross-Validation

SMOTE is applied to mitigate the issue of the unbalanced data labels to enhance the generalize efficiency of the classification model for detecting αT carriers. Then, we reassess them after using the same classifiers and 10-fold cross-validation. The findings from this experiment are presented in Table 9. GBC outperforms with 93.46% accuracy, 95.46% recall, and 93.65% f-score on first dataset. XGB plus feature importance using GBDT gives 83.33% accuracy with second dataset.

### 5.4. Experiment IV: Classification with Normalization, SMOTE, Feature Selection, and 10-Fold Cross-Validation

Data normalization added in above scenario of involving resampling technique with selected features and 10-fold validation. RF gives the highest accuracy of 92.79% with recall 93.89% and 92.72% with EFS, as experiment result of first dataset shown in Table 9b. ADA shows the highest accuracy of 90% (Table 11) for the second dataset with feature importance using GBDT (Hb, PCV, MCV, MCH, MCHC, RDW, WBC, Neut, Lymph, PLT, HBA2, and HBF).

Accuracy of both datasets is compared side by side in Table 10 with and without feature selection. Results shows signification improvements such as normalization, SMOTE and 10-fold validation applied in combination with feature selection for evaluation in Table 11. Results of our model on both datasets are also compared with techniques presented by other researchers in Table 12.

**Table 12:** A performance comparison of existing techniques with our proposed method (NA means data not available).

Ref.	Method	Features	Accuracy
[45]	CNN, SVM, MLP, RF, PLS, LR, ET, LGBM, XGB, DT, and KNNs implemented in Python	Age, Gender, MCHC, RDW, Hct, MCV, RBC, Hb, MCH	80.77% with CNN
[40]	KNN, SVM, LR, NB, MLP, RF, ADA Boosting, Xgboost, DT, and GBC using Google Colab Data Balance: SMOTE	TLC, MCV, MCH, RDW, MCHC, PCV, RBC count, HGB, PLT, age, gender	100% with ADA
[51]	SVM.	Hb, WBC, HCT, RBC, MCH, MCV, RDW, PLT, MCHC RDW, RDWI, E&F, S&L, G&K, MDHL, MCHD, HH index, αT, βT	NA
[52]	RF with R software version 3.6.2.	RDW, Hct, MCV, RBC, Hbf, Hba, Hb, MCH, pregnancy and age,	91.5%
[58]	DNN model with 11 features then removing some of the 11 features	HB, RBC, RDW MCV, HCT, WBC, MCH, PLT, MCHC, patient’s sex and age but RDW, age, sex, WBC, and PLT are more important	89.7%
Our Model (First dataset)	LR without feature selection	Age, Sex, Hct, Hb, MCH, MCV, MCHC, RDW, RBC count	88.31%
	χ2 feature section with LR Classifier	MCV, MCH, RDW, Hb, RBC count, and Hct	91.56%,
	SMOTE, Feature importance base selection, and GBC	Age, Hb, MCV, MCH, RDW, RBC count	93.46%
	Normalization, SMOTE, Exhaustive Feature Selection with RF	Age, Sex, Hb, MCV	92.79%
Our Model (Second dataset)	KNN (*n* = 3) without feature selection	Hb, PCV, RCB, MCV, MCH, MCHC, RDW, WBC, Neut, LYMPH, PLT, HBA, HBA2, HBF, Sex	82.35%
	Exhaustive Feature Selection (EFS) with LR classifier	HB, PCV, RBC, MCV	88.33%
	SMOTE, Feature importance base selection, and XGB	Hb, PCV, RBC, MCV, MCH, WBC, Lymph, HBA2	88.33%
	Normalization, SMOTE Feature importance using GBDT with ADA	Hb, PCV, MCV, MCH, MCHC, RDW, WBC, Neut, LYMPH, PLT, HBA2, HBF	90.0%

## 6. Discussion

The first section of this paper includes a systematic review of AI-based and ML-based thalassemia diagnostic methods. This analysis focuses on five particular topics: databases (Table 1), data preparation (Table 2), the classification of thalassemia and health threats using machine learning (Table 3), management applications (Table 4) based on ML, and measures of performance for evaluating the effectiveness of the classification model (Table 5). The analysis of data preparation, normalization, feature selection, and ML classification approaches is covered in the second section of the study with a working example. Nine ML basic classifiers are used with two public datasets from Kaggle in the experiment. The performance evaluation parameters for determining the categorization of αT include accuracy, recall, and F1-score. In addition, feature selection is beneficial; however, it has certain issues. These issues include (i) the complexity of time and (ii) the automatic determination of the optimum range of attributes. A simple feature selection with minimal time complexity is used to solve these issues.

Without feature selection in the first dataset, the LR classifier can achieve its maximum accuracy of 88.31%, recall of 88.19%, and f-score of 88.02%. Six features chosen by χ2 have helped LR achieve maximum accuracy of 91.56%, 91.04% recall, and 92.65% F1-score. SMOTE improves overall performance as GBC outperforms with 93.65% F1-score, 95.46% recall, and 93.46% accuracy with 10-fold. However, normalization reduces the effectiveness of resampling techniques that use selected features with 10-fold validation. The maximum accuracy is provided by RF, with accuracy of 92.79%, recall of 93.89%, and EFS of 92.72%. However, the outcome is still superior to the first two experiments. Table 9 shows that the suggested model, when combined with SMOTE and 10-fold cross-validation, achieved good classification accuracy. The second dataset KNN (*n* = 3) achieved 82.35% accuracy, 62.32% f-score, and 61.11% recall with all attributes. Only LR shows higher accuracy of 88.23% with four features (Hb, PCV, RBC, and MCV) chosen by Exhaustive Feature Selection (EFS). After applying normalization and SMOTE, ADA gives maximum accuracy of 90%. To emphasize the success of the approach even more, the comparative findings are provided in Table 12. Comparing the many cutting-edge methodologies, our model generated the highest accuracy. All of the models on the list have an average of nine features and an accuracy range of 80.77% to 100%. Two models employ a variety of AI and ML strategies; one achieved an accuracy of 80.77% using CNN, [45] and the other gave 100% accuracy by combining data balancing methodology. SMOTE is followed by ADA [40]. The next three approaches each employ a single classifier; the first uses SVM [51], the second uses RF [52] while achieving an accuracy of 91.5%; and the third employs a different form of DNN, which achieves an accuracy of 89.7%.

In contrast to our suggested methodology, only one employs SMOTE [40] for imbalanced data when it comes to preprocessing, data balancing or feature selection. All methods employ between nine and sixteen features; however, our feature selection process only uses a maximum of six, with an accuracy rate that is greater than the majority of the strategies described. As in Table 7, feature selection enables 91.5% accuracy to be attained with fewer characteristics. Normalization, SMOTE, and 10-k cross-validation are used to further enhance the result, which increased from 92.79% to 93.46%. You can see that different classifiers employ varying numbers of features in the results as diverse feature selection techniques are used in combination with nine classifiers. The minimal number of TT participants is a drawback of our study. Therefore, it is feasible that other researchers may make hypotheses utilizing this unique approach and make an effort to refute our findings in regions where they are more prevalent. Comparable studies, however, lacked a control group, and the bulk of studies [7,46,47,48,49,50,59,63] similarly paid little attention to the group sizes we believed to be equal. As a consequence, we think that our study offers a more accurate evaluation.

## 7. Conclusions

This paper aims to investigate the influence of feature selection methods on the precision of thalassemia predictions. Experiments were conducted using all features to discern the impact of feature selection on performance, followed by a selected subset of features. Nine classification algorithms were assessed: KNN, DT, GBC, LR, ADA, XGB, RF, LGBM, and SVM. The effectiveness of the model was measured using accuracy, F1-score, and recall metrics. Our experimental results emphasize the strength of the proposed method in pinpointing carriers of αT. Without feature selection, the peak accuracy achieved was 88.31%, which improved to 91.56% when the χ2 feature selection methods were employed in conjunction with the LR classifier by using the first dataset. For the second dataset, accuracy was improved to 88.23% EFS, and LR from 82.35% was achieved from KNN (*n* = 3). Additionally, our findings indicate that oversampling with SMOTE, RFE, and 10-fold validation effectively enhances the detection rate of αT carriers. Notably with the first dataset, the GBC classifier stands out, delivering 93.46% accuracy, 93.89% recall, and 92.72% F1-score. Maximum accuracy of 90% showed by ADA in conjunction with SMOTE, feature importance using GBDT, and 10-fold validation for the second dataset. For optimal performance of the model, comparing various feature selection strategies and classifier combinations, is imperative. However, predicting which combination will be most effective without extensive experimentation, and analysis is challenging. Future works will consider devising hybrid algorithms that adopt multiple feature selection techniques to extract the richest feature subsets. Additionally, leveraging real-time medical datasets from thalassemia patients could further enrich the model structure.

## Data Availability

Publicly available datasets were analyzed in this study. This data can be found here: [https://www.kaggle.com/code/plenoi/thalassemia-deep-07072022/input?select=Alpha-2_addition-230622.xlsx] (accessed on 5 July 2023).

## Abbreviations

**Acronyms**	**Meaning**
αT	Alpha-thalassemia
ADA	AdaBoost
ANFIS	Adaptive neuro-fuzzy inference system
ADASYN	Adaptive Synthetic
ANN	Artificial neural network
AUC	Area Under the ROC Curve
βT	Beta-thalassemia
BLTREED	Bayesian Logit Treed
CBC	Complete Blood Count
CNN	Convolutional Neural Network
CNN-LSTM	CNN Long Short-Term Memory Network
CRISPR	Clustered Regularly Interspaced Short Palindromic Repeats
DSIFT	Dense Scale Invariant Feature Transform
DTL2	CNN as a feature extractor
DT	Decision Tree
DHS	Dynamic Harmony Search
E&F	England and Fraser
ETC	Extra Tree Classifier
ET	Extremely Randomized Trees
ELM	Extreme Learning Machine
FNR	False-negative rate
FPR	False-positive rate
GBM	Gradient Boosting Machine
GBC	Gradient Boosting Classifier
GBDT	Gradient Boosting Decision Trees
G&K	Green and King
GRU	Gated Recurrent Unit
Hb/HBA	Hemoglobin
HBA2	Hemoglobin A2
HBF	Fetal hemoglobin
HGB	Hemoglobin in grams per deciliter of blood
HH index	Huber–Herklotz index
IDA	Iron deficiency anemia
IoU	Intersection Over Union
KNN	K-Nearest Neighbors
LR	Logistic Regression
LSTM	Long Short-Term Memory
LGBM	Light Gradient Boosting Machine
L-SVC	Liner SVC
lgbmR	Light GBM Regressor
Hct	Hematocrit
MCV	Mean Cell Volume
MCH	Mean Cell Hemoglobin
MCHC	Mean Cell Hemoglobin Concentration
MCHD	Index, Mean Cell Hemoglobin Density
MDHL	Mean Density of Hb/liter of blood
MLPs	Multilayer perceptron
MLR	Multiple Linear Regression
NB	Naive Bayes
NCA	Component Analysis Feature Selection
NN	Neural Networks
NPV	Negative predictive value
Neut	Neutrophils
PBIS	Pattern-based index selection
PCA	Principal component analysis
PCV	Polycythemia vera
PLT	Platelet
PTPP	Punjab Thalassemia Prevention Program
PPV	Positive predictive value
PLS	partial least squares
RANSAC	Random sample consensus
RF	Random Forest
RBC	Red blood cell
RBF	Gaussian radial basis function
RDW	Red Cell Distribution Width
RDWI	Red Cell Distribution Width Index
RELM	Regularized Extreme Learning machine
SVD	Singular Value Decomposition
SVC	Support Vector Classifier
SVM	Support Vector Machine
S&L	Shine and Lal
SMOTE	Synthetic Minority Oversampling Technique
TSVM	Twin Support Vector Machines
TT	Thalassemia Trait
WBC	White blood cell
XGB	Extreme Gradient Boosting
Xgboost	Extreme Gradient Boosting
χ2	Chi-square
Iχ2	Iterative Chi-Square
